# Supplementary material for: Psychological Resilience and Health-Related Quality of Life in 418 Swedish Women with Primary Breast Cancer: Results from a Prospective Longitudinal Study
Source: Cancers (Basel). 2021 May 6;13(9):2233. doi: 10.3390/cancers13092233 (PMC8125618; doi:10.3390/cancers13092233)
Supplement: Supplementary file 1 [file cancers-13-02233-s001.zip › cancers-1173091-supplementary.pdf]

cancers

Supplementary Materials: Psychological Resilience and Health-Related Quality of Life in 418 Swedish Women with Primary Breast cancer: Results from a Prospective Longitudinal Study

Åsa Mohlin, Pär-Ola Bendahl, Cecilia Hegardt, Corinna Richter, Ingalill Rahm Hallberg and Lisa Rydén

Table S1. Summary of clinicopathological and demographic characteristics for included and excluded participants, and additional participant characteristics.

Variables		Baseline Study at Diagnosis (*n* = 517)		Follow-Up Study at One Year Post Diagnosis (*n* = 418)		Excluded Participants (*n* = 99)	
Psychological resilience (CD-RISC25)		Mean (SD): 70.6 (12.7)		Mean (SD): 68.9 (14)		Mean (SD): 71 (11.2)	
Age (years)		Mean (SD): 62 (11) Median: 64		Mean (SD): 62 (11) Median: 64		Mean (SD): 62 (12) Median: 63	
		n	%	*n*	%	*n*	%
Study site	Halmstad	163	31	135	32	28	28
	Helsingborg	30	6	20	5	10	10
	Karlskrona	149	29	120	29	29	29
	Växjö	175	34	143	34	32	32
Menstrual status	Premenopausal	93	19	74	18	19	19
	Postmenopausal	397	81	325	78	72	73
	Unknown	27		19		8	
Mode of detection	Screening	334	65	269	64	65	66
	Symptomatic	182	35	148	35	34	34
	Unknown	1		1		0	
Stage	0	29	8	24	6	5	5
	I	335	52	272	65	63	64
	II	148	32	117	28	31	31
	III	3	8	3	1	0	
	Unknown	2		2		0	
Type of cancer	Carcinoma *in situ*	46	9	36	9	10	10
	Invasive cancer	455	91	369	91	86	90
	Unknown	16		13		3	
Tumor size	≤ 20mm	332	73	271	73	61	71
	> 20mm	124	27	99	27	25	29
	Unknown	61		48		13	
Histological type	Ductal	361	80	296	81	65	76
	Lobular	49	11	38	10	11	13
	Mixed/other	42	9	32	9	10	12
	Unknown	65		52		13	
Estrogen receptor (ER) status	Positive	224	88	194	89	30	86
	Negative	32	13	27	12	5	14
	Unknown	261		197		64	
Progesterone receptor (PR) status	Positive	182	71	155	70	27	75
	Negative	75	29	66	30	9	25
	Unknown	257		197		63	
Human epidermal growth factor receptor 2 (HER2) status	Positive	48	11	42	12	6	7
	Negative	398	89	319	88	79	93
	Unknown	71		57		14	

Ki67 (%)	≤20%	228	51	188	52	40	47
	>20%	218	49	173	48	45	53
	Unknown	71		57		14	
Primary therapy	Surgery	491	95	396	95	95	96
	Systemic therapy	26	5	22	5	4	4
Axillary surgery	Yes	508	98	410	98	98	99
	No	9	2	8	2	1	1
Social network	Living alone	114	22	85	20	29	29
	Living with child/children < 18 years old only	11	2	5	1	6	6
	Living with adult/adults and child/children < 18 years old	65	13	53	13	12	12
	Living with adult/adults only	327	63	275	66	52	53
Educational level	Primary school < 9 years	70	14	55	13	15	15
	Primary school completed	74	14	52	12	22	22
	Upper secondary education	92	18	84	20	8	8
	Post-secondary education < 2 years	66	13	39	9	27	27
	Post-secondary education ≥ 2 years	204	39	182	44	22	22
	PhD (doctoral education)	11	2	6	1	5	5
Financial situation	Able to pay an unexpected bill of SEK 11.000/ EUR 1100	466	90	378	90	88	89
	Unable to pay an unexpected bill of SEK 11.000/ EUR 1100	51	10	40	10	11	11
